# Slowing Down Ageing: The Role of Nutrients and Microbiota in Modulation of the Epigenome

**DOI:** 10.3390/nu11061251

**Published:** 2019-06-01

**Authors:** Agnieszka Gadecka, Anna Bielak-Zmijewska

**Affiliations:** Nencki Institute of Experimental Biology, Polish Academy of Sciences, 3 Pasteur St., 02-093 Warsaw, Poland; a.gadecka@nencki.gov.pl

**Keywords:** ageing, epigenetic, nutrition, microbiome

## Abstract

The human population is getting ageing. Both ageing and age-related diseases are correlated with an increased number of senescent cells in the organism. Senescent cells do not divide but are metabolically active and influence their environment by secreting many proteins due to a phenomenon known as senescence associated secretory phenotype (SASP). Senescent cells differ from young cells by several features. They possess more damaged DNA, more impaired mitochondria and an increased level of free radicals that cause the oxidation of macromolecules. However, not only biochemical and structural changes are related to senescence. Senescent cells have an altered chromatin structure, and in consequence, altered gene expression. With age, the level of heterochromatin decreases, and less condensed chromatin is more prone to DNA damage. On the one hand, some gene promoters are easily available for the transcriptional machinery; on the other hand, some genes are more protected (locally increased level of heterochromatin). The structure of chromatin is precisely regulated by the epigenetic modification of DNA and posttranslational modification of histones. The methylation of DNA inhibits transcription, histone methylation mostly leads to a more condensed chromatin structure (with some exceptions) and acetylation plays an opposing role. The modification of both DNA and histones is regulated by factors present in the diet. This means that compounds contained in daily food can alter gene expression and protect cells from senescence, and therefore protect the organism from ageing. An opinion prevailed for some time that compounds from the diet do not act through direct regulation of the processes in the organism but through modification of the physiology of the microbiome. In this review we try to explain the role of some food compounds, which by acting on the epigenetic level might protect the organism from age-related diseases and slow down ageing. We also try to shed some light on the role of microbiome in this process.

## 1. Introduction

Ageing is defined as a continuous accumulation of detrimental changes in the whole organism due to the impaired functioning of many tissues and organs. A constant loss of function of the muscle and skeletal systems, memory disabilities and a higher vulnerability to, for example, infections, are observed with age [1]. Age is the main risk factor for certain diseases, called age-related diseases (ARD). Ageing is associated with chronic low grade inflammation, which is probably both the cause and the result of increased age [2]. Because the population of people over 65 years old is increasing very quickly, it is becoming necessary to develop some new approaches to improve their quality of life. It is generally accepted that it is much more reasonable to protect from ageing than to treat a particular disease, especially given that age-related diseases usually arise jointly, which entails the need to take many different medicines that very often act antagonistically [3]. To slow down ageing and to reduce or postpone ARD, well-defined and efficient strategies must be developed. These strategies should preferably be compatible with our lifestyle and habits. Such an attitude should not be complicated and inscribed in a normal everyday functioning. Currently, nobody needs to be persuaded that a daily diet is one of the most important elements of our health and wellbeing. The existing data clearly show that what we eat and how much we consume determines our healthspan. One of the most convincing examples is the population of Okinawa Island, on which the number of centenarians (people over 100 years of age) is the highest in the world. The diagnosed reason of such a long lifespan is a lower calorie consumption [4,5].

The observations of some beneficial effects exerted by a diet are supported by a great deal of evidence from scientific studies. Certain mechanisms of such an impact have been already defined and certain molecular factors have been recognized. It has been proven that the ageing process is malleable, and that by appropriate proceedings, we can influence the pace and the course of ageing and ARD [6]. However, currently, the most appreciated anti-ageing intervention is caloric restriction or certain approaches mimicking it [7]. To such activities belong: a strongly defined lifestyle consisting of a proper healthy diet, mild physical activity, and the avoidance, in the broad sense, of stress. Since it is already well recognized that ageing can be modified, our aware attitude should help to elongate our healthspan and possibly also the lifespan.

It has been documented that one of the most recognized causes of ageing is cellular senescence. Firstly, it has been observed that senescent cells accumulate with ageing in many tissues [8,9,10] and later on, it has been proved that elimination of such cells improves the functioning of the organism and slightly elongates the lifespan of model animals [11,12,13]. A reduction of the number of senescent cells was achieved mostly in genetically modified animals, which is not possible to perform in humans. The non-genetic eradication of senescent cells is not easy but recent studies have shown that it is possible. A new approach, named senotherapy, is being intensively developed and some promising results, which can be transferred to humans, were obtained [14].

One of the most spectacular and significant changes observed in senescent cells is modification of the chromatin structure [15]. The role of the genotype in ageing and longevity is an important issue, however, a growing evidence has shown that the role of epigenetics cannot be neglected and can be equally or, in some situations, even more important than the genetic profile. Epigenetic changes can be influenced by a variety of environmental factors [16,17,18,19]. Functional foods and nutraceuticals seem to be among those most important [19] as they can promote health and longevity and prevent ARD [20]. Lower global chromatin compaction and locally increased condensation lead to an elevated vulnerability of the cell nucleus to destruction, and may also modify gene expression. This is due to epigenetic and post-translational modifications of DNA and histones, respectively. Acquired epigenetic marks are inherited by offspring, which clearly proves their significance. Moreover, manipulation of epigenetic modifications seems to be less complicated and does not raise any ethical doubts as does genetic intervention. Nutrients can modify epigenetic marks; however, the impact of diet on chromatin may not always be direct. There are some suggestions that the influence can be mediated by the microbiome [17]. The composition of the gut bacteria depends on the age and it varies in health and disease [21]. The elderly population very often suffers from detrimental changes of several biological functions, which are associated with intestinal microbiota rearrangement. It has been proven that nutrients are involved in the shaping of the microbiota composition, which gives a promise that a well-composed diet can alleviate some age-related disabilities. The role of the microbiome in ageing is intensively studied and it is believed that the composition and the quality of the microbiome can determine healthspan and longevity [22]. The links between the diet, the microbiome and chromatin structure will be discussed in this review.

## 2. Ageing, Cellular Senescence and Anti-Ageing Interventions

Ageing is associated with some features, which are characteristic for all elderly; however, age-related diseases do not affect all of them equally. To common age-related health problems belong: impaired visions and hearing, sarcopenia, osteoporosis, increased vulnerability to infections, impaired wound healing and decreased skin elasticity. With age increases the risk of appearance of ARD, which include: neurodegenerative diseases (e.g., Alzheimer’s and Parkinson’s diseases, AD and PD, respectively), cardiovascular diseases (CVD, hypertension and atherosclerosis), type II diabetes, lung diseases (chronic obstructive pulmonary disease, COPD and idiopathic pulmonary fibrosis, IPF), osteoarthritis, cataract, glaucoma and certain types of tumors (e.g., breast, colon, lung cancer) [1]. It has been documented that there is increased number of senescent cells in tissues and organs affected by all age-related diseases [1,23,24,25,26,27,28]. For this reason, the role of cellular senescence in ageing and ARD cannot be neglected. Therefore, cellular senescence is intensively studied and there are examples showing that an improvement of organismal functioning is associated with the alleviation of the senescent phenotype [29] and vice versa, the eradication of senescent cells is followed by a reduction of certain age-related disabilities [11,12].

Cellular senescence is not always detrimental, as it may appear based on its engagement in ageing and ARD. The function of this basic cellular process is complex and the relevance of senescence depends on the age of the organism [30]. Senescence is essential for proper body shaping during embryonic development [31,32]. In a young organism it serves a beneficial function in tissue regeneration, in protection from fibrosis [33,34] and as a barrier from cancer (cessation of proliferation of damaged cells) [30]. However, in an old organism the number of senescent cells increases. They are involved in the generation of a chronic low-grade inflammatory state via the excessive secretion of certain proteins (senescence-associated secretory phenotype, SASP), and an enhanced production of reactive oxygen species (ROS). Such properties of senescent cells lead to microenvironmental changes supporting tumor progression, lowering of the regenerative potential and the renewal of tissues, and to senescence of neighboring non-senescent cells.

Cellular senescence can occur as a result of telomere shortening, and is then called replicative senescence, or as a response to stress and is then termed stress-induced premature senescence (SIPS) [35,36]. Senescence can be induced by intrinsic (ROS production, metabolic malfunctions, oncogene overexpression, DNA damage, ER-stress, and by genetic and epigenetic changes e.g., chromatin structure dysfunction) or extrinsic (exposition to physical or chemical factors) stimuli [37,38].

Cellular senescence is listed as one of the nine cellular hallmarks which contribute to ageing: genomic instability, telomere attrition, epigenetic alterations, stem cells exhaustion, altered intercellular communication, cellular senescence, mitochondrial dysfunction, loss of proteostasis, deregulated nutrient sensing [39]. However, all of the mentioned features are strongly related to cellular senescence. Either they can lead to cell senescence (genomic instability, telomere attrition, epigenetic alterations, mitochondrial dysfunction and loss of proteostasis) or are the result of senescence (stem cell exhaustion, altered intercellular communication, deregulated nutrient sensing).

**Figure 1:**
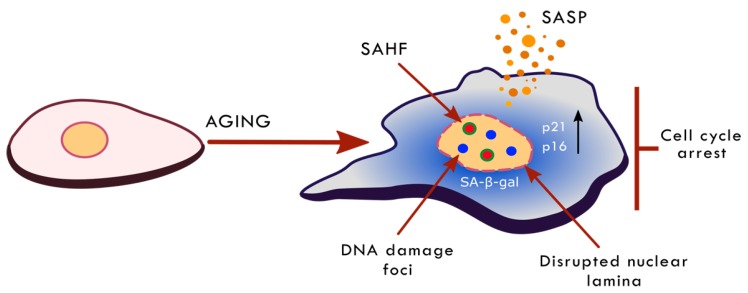
Global changes and markers of senescent cells. During ageing, the accumulation of senescent cells is often observed. Senescent cells are characterized by permanent cell cycle arrest, increased size, elevated activity of senescence associated β galactosidase (SA-β-gal) and higher levels of cell cycle inhibitors, p16 and/or p21. Moreover, senescent cells secrete various proteins such as cytokines, growth factors and proteases. This phenomenon is generally referred as senescence associated secretory phenotype (SASP). Nuclear changes are manifested by disrupted structure of nuclear lamina (for instance, due to downregulation of Lamin B1), local condensation of chromatin in the form of senescent associated heterochromatin foci (SAHF) and by DNA-SCARS (DNA segments with chromatin alterations reinforcing senescence), which form in response to DNA damage.

Senescent cells are characterized by multiple alterations in cell morphology, physiology and biochemistry (summarized in Figure 1). The first most important and essential feature of senescence is the permanent cessation of proliferation. However, senescence concerns not only the proliferation-competent cell but also post-mitotic cells, such as neurons, skeletal muscle cells and cardiac myocytes [40]. The most recognized senescence markers include: increased activity of a lysosomal enzyme, senescence-associated β-galactosidase (SA-β-gal), increased level of cell-cycle inhibitors (p21, p16), increased frequency of DNA damage and activation of the DNA damage response (DDR) pathway, increased the production and secretion of SASP components (a bystander effect is senescence induction in neighboring cells) and changes in chromatin structure and gene expression [33,41]. Until now, there was no universal marker of cell senescence. The most widely accepted reason of cellular senescence is DNA damage [38,42,43]. However, there are some examples showing that cells undergo senescence without DNA breaks [38,40,44]. Currently, there is an assumption that senescence is a response to the widely understood stress conditions. There are some examples to show that cell senescence may be the result of reticular stress, nucleolar stress, chromatin changes, the activation of the DDR pathway without DNA damage, and osmotic stress [38].

Studies on the mechanism of ageing and senescence have led to the development of certain anti-ageing approaches, which have proved their worth on animal models. Some of the most promising approaches include caloric/diet restriction (CR/DR), which reduces the calorie intake without causing malnutrition, and some activities mimicking it, such as certain micro-nutrients or mild physical activity [45,46]. Dietary restriction has been shown to be effective in several species (yeast, fruit flies, nematodes, rats, dogs, primates) [7,47,48,49]; however, it is difficult to apply it to humans. Nonetheless, a similar approach, namely intermittent fasting (IF), gave comparable results [50,51]. The effects of CR are related not only to lifespan extension but, first of all, to healthspan improvement, that is alleviation of neurodegeneration, sarcopenia, cardiovascular and metabolic diseases, and decreased incidence of cancer [52,53,54]. In humans, short-term CR increased multiple markers of metabolic and cardiovascular health [55]. There are some suggestions concerning the beneficial effects of the mechanism involved in CR. The role in CR is ascribed to a group of enzymes involved in the regulation of many cellular processes indispensable for maintaining homeostasis, namely sirtuins, reviewed in [7]. Sirtuin 1 and 6 (SIRT1 and 6) are strongly involved in the modulation of chromatin structure by histone deacetylation and have an impact on DNA methylation. The level of almost all sirtuins is elevated due to CR [56]. On the other hand, the expression and activity of these enzymes decrease with age [7,44,57]; moreover, SIRT1 and 3 were suggested as markers of frailty, one of the most characteristic features associated with ageing [58].

The main signalling pathways involved in longevity, and thus proposed to be a target for ageing intervention, include mTOR–S6K signalling and the GH/IGF-1 axis, which require AMPK and specific sirtuins [54]. They are mutually related and regulated. Inhibition of mTOR and IGF-1, or its receptor, is a recognized approach to elongate the lifespan, reviewed in [7]. The mTOR–S6K pathway is inhibited by AMPK, which regulates, and is regulated by sirtuins. SIRT1, in turn, is involved in the p53 pathway, which antagonizes IGF-1 signalling [59].

## 3. Epigenetics and Nuclear Landscape; Ageing-Associated Changes of Chromatin Structure

The genetic material of all Eukaryotes is confined to a highly organized nucleus measuring only a few microns. To fit in such a restrained volume, 146 kb long segments of double stranded DNA are wound on histone octamers forming the basic structure of a nucleosome. The nucleosomes are then further condensed by linker histones and scaffold proteins into higher order chromatin. The chromatin is enclosed by a double nuclear membrane spiked with complexes of transmembrane proteins—nucleoporins. The interior membrane is lined with a mesh of intermediate filaments, namely lamins, that constantly interact with chromatin domains [60,61].

Epigenetics is defined as the study of heritable changes in gene function that do not entail any alteration in DNA sequence [62]. The epigenome acts as a molecular link between the genome and the environment. The environmental impact is related to the lifestyle, including diet, physical activity, stressors, chemical exposition and addictions such as smoking and drug abuse. All these factors are able to alter the epigenetic landscape and favor ageing-related disease phenotype. The epigenetic mechanisms include: DNA methylation, histone modifications and post transcriptional regulation of gene expression by non-coding RNA (microRNA, miRNA) [63]. These processes are able to regulate synergistically and cooperatively gene expression by changing chromatin organization and DNA accessibility. Although epigenetic marks are to a great extent inherited, the epigenetic landscape is not given once and for all. It is constantly prone to perpetual fluctuations throughout the lifespan and is profoundly altered in ageing organisms. The resulting gene expression profile, however, can be relatively easily modified by pharmaceuticals [64], physical exercise [65], diet [66] and even gut microbiota [67].

### 3.1. DNA Methylation

One of the prominent examples of epigenetic modification is DNA methylation, which is characterized by covalent transfer of methyl group from S-adenosylmethionine (SAM) to the fifth position of cytosine ring (5mC) within cytosine-phosphate-guanine (CpG) dinucleotide. DNA methylation in vertebrates is mainly restricted to CpG sites (CpGs) and 60–80% of CpGs in the human genome are methylated. Approximately 7% of CpGs are located in CpG islands (CGIs), which are regions of high CG density [68]. Depending on the number of methylated cytosines within the site, DNA can be either hypo- or hypermethylated. Hypermethylation of CpG islands is associated with transcriptional repression and gene silencing whereas hypomethylation with transcriptional activation [69]. This often applies CpG islands (CGIs) in promoter regions of particular genes. Approximately 60–70% of genes have a CpG island within their promoters [70]. Although these regions are considered mostly unmethylated [71], this state changes drastically in pathological conditions like cancer, or most importantly, during ageing. As the organism is getting older, hypermethylation of tissue specific genes or genes encoding transcription factors is observed [72,73] in contrast to global loss of methylation [74,75], leading to drastic changes in gene expression profile as compared to young controls. This phenomenon has found its application in several recently established ‘epigenetic clocks’, where biological and chronological age can be estimated on the basis of DNA methylation level [76,77].

In humans, four DNA methyltransferases (DNMT) genes, namely *DNMT1*, *DNMT2*, *DNMT3A*, and *DNMT3B* have been identified [78]. Three of them are classic methyltransferases (*DNMT1*, *DNMT3A*, *DNMT3B*) and the function of DNMT2 is a little controversial. Moreover, DNMT-like regulatory enzyme DNMT3L (DNA methyltransferase 3 like) is worth to be mentioned. DNMT1 was the first methyltransferase identified and is responsible for replicating the methylation pattern on newly synthesized DNA strands based on the hemi-methylated template strand, hence it plays a maintenance role. On the other hand, DNMT3A and DNMT3B are believed to methylate DNA de novo. Nevertheless, recent studies have suggested that these three enzymes can play complementary roles in mammals [79]. As far as DNMT3L is considered, it cannot be classified as a typical methyltransferase since it is catalytically inactive. Instead, it works as a cofactor in the regulation of methyltransferase activity by forming a complex with histone H3 that is unmethylated on lysine 4, to recruit DNMT3A and perform de novo DNA methylation [80]. Last but not least, DNMT2 shares a strong homology with other DNA methyltransferases although its methyltransferase activity is thought to be marginal [81]. It is mainly considered to play a leading role in tRNA methylation [82]. However, recent studies suggest a putative contribution of *DNMT2* to DNA methylation. Khalil et al. show that the activity of DNMT2 in aged mouse macrophages is considerably increased, which leads to hypermethylation in promoter regions of autophagy genes *Atg5* and *LC3B*. This results in reduced autophagy, which is one of the causes of chronic inflammation, a characteristic feature of aged organisms [73]. Moreover, *DNMT2* is shown to be upregulated in replicatively senescent human fibroblasts, which suggests its role in longevity regulation. Interestingly, silencing of DNMT2 results in changes in proliferation-related and tumor suppressor miRNAs level and leads to proliferation inhibition and induction of cellular senescence mediated by oxidative stress [83]. *DNMT2* silencing in mouse fibroblasts leads to, inter alia, telomere shortening, elevation of cell cycle inhibitors and DNA damage, resulting cell senescence [84]. It is believed that DNA demethylation is not only a passive process occurring as a result of the lack of DNMT1 but can be achieved by active demethylation [19]. The methylated cytosine is oxidized to 5-hydroxymethylcytosine (5hmC) by the ten-eleven translocation (TET) enzymes consisting of three family members, i.e., TET1, TET2 and TET3 [85]. These proteins can catalyze further 5hmC oxidation to 5-formylcytosine (5fC) and 5-carboxylcytosine (5caC), that usually ends up with the removal of the modified base by base excision repair or decarboxylation [86]. How the process of DNA demethylation proceeds in vivo, however, is still under extensive investigation. Nevertheless, different tissues seem to accumulate 5hmC at varying levels [87,88], and the enrichment is usually observed at promoters of specific genes [89]. This indicates, that 5hmC does not only serve as an intermediate in the active DNA demethylation but can also stand as an epigenetic regulatory mark controlling gene expression. The 5hmC is most abundant in embryonic stem cells, adult somatic stem cells and brain tissue [88,89] although localization of the 5hmC-enriched regions depends on the type of cell and developmental stage. Profound changes are found in ageing mouse brains; a study revealed a global increase in hippocampal 5hmc content, which was unrelated to oxidative stress [90]. The same trend was noted in substantia nigra, where the increase of 5hmC was observed in contrast to striatum which has stable DNA methylation status across ageing [91].

Moreover, chromatin accessibility is regulated via a crosstalk between DNA methylation and histone modifications. Methylated DNA recruits histone deacetylases and histone methyltransferases e.g., SuV39H1 which, by methylating H3K9 (histone H3 lysine 9), tightens the chromatin structure [19]. Moreover, HP-1 (heterochromatin protein 1) is responsible for recruitment of DNA methyltransferases, DNMTs [92].

### 3.2. Posttranslational Modification of Histones

The next level of nuclear organization and gene expression control concerns chromatin structure predominantly controlled by posttranslational modifications (PTMs) of histones. Histones are highly conserved DNA-binding proteins, that form a nucleosome core. Each nucleosome consists of two copies of canonical histones H2A, H2B, H3 and H4. The double stranded DNA is wound on the established histone octamer and the whole complex is stabilized by linker histone H1 [93]. Each core histone (except for H4) has variants which differ in amino acid sequence and can be synthesized independently of DNA replication both in mitotic and post-mitotic cells [94]. The incorporation of histone variants certainly adds up to the complexity of the nucleosome organization and gene expression control.

Histones can be post-translationally modified on the N-terminal tails protruding from the globular histone core. Depending on the type of modification the impact on chromatin accessibility and stability differs considerably. Among the plethora of PTMs the most prevalent are methylation, acetylation, phosphorylation and ubiquitination [95]. These modifications are precisely controlled by highly specialized “writers”, “erasers” and “readers”, that incorporate, remove and recognize PTMs in histones. Writers include histone acetyltransferases (HATs), that transfer an acetyl group from acetyl-CoA on specific lysine residues in the histone tail [96], and histone methyltransferases (HMTs) that can mono-, di- and tri-methylate lysine residues [96]. Acetylation of histones H3 and H4 leads to an open and active chromatin structure. On the other hand, histone methylation can both activate and repress gene expression. Trimethylation of K4, K36 and K79 in histone H3 results in active transcription, while trimethylation of K9, K27 and K20 is associated with silenced chromatin [97]. The trimethylation of histone H3 at lysine 9 (H3K9me3) is catalyzed by the Suv39H1 methyltransferase. This HMT can directly interact with HP-1 and regulates chromatin compaction [98]. Erasers, such as histone deacetylases (HDACs and Sirtuins) and histone demethylases (HDMs), counteract the action of HATs and HMTs and modify chromatin condensation and gene expression. Readers, on the other hand, are proteins that possess domains with high affinity for the modified histone sites to direct a particular transcriptional outcome [99]. Domains that recognize methylated histones include chromo, PHD, WD40, Tudor, MBT domains and more, while histone acetylation is recognized by bromo domains and tandem PHD domains. Interestingly, readers can target multiple PTMs within a histone, nucleosome and even multiple nucleosomes and their action can be controlled by non-coding RNAs. Moreover, readers can recruit factors that are involved in transcription, replication or DNA damage repair [100].

With the help of histone modifiers chromatin can adopt two dynamic conformations: that of transcriptionally inactive heterochromatin, condensed during the interphase and replicated in the late S phase, and that of euchromatin accessible to transcription, decondensed during the interphase and replicated in the early S phase [101]. Heterochromatin can be further divided into constitutive heterochromatin (telomeres, pericentromeric regions) rich in H3K9me3, protein HP1 (which stabilizes chromatin compaction) and H4K20me3, and facultative heterochromatin, characterized by high H3K27me3 level, which arises temporarily in euchromatic regions and silences individual genes. On the other hand, euchromatin is usually abundant in active chromatin markers, for example H3K4me3, H3K36me3 and acetylated histones H3 and H4 [102].

### 3.3. Sirtuins

Sirtuins, through histone deacetylation, take part in the formation of constitutive and facultative heterochromatin. The most studied sirtuins are SIRT1 and 6. SIRT1 is involved in deacetylation of lysine K9 in histone 3 (H3K9), which leads to its trimethylation (acetylation of K9 protects it from trimethylation) and facilitates the interaction with heterochromatin protein 1 α (HP1α). Such interaction keeps the chromatin in a closed state resulting in gene silencing [103,104]. SIRT1 preferentially deacetylates H4K16, H3K9, H3K56 H3K14, H1K26 and H1K9 [104,105]. Moreover, this enzyme influences chromatin condensation by regulating histone expression and the level and activity of some histone modifying enzymes, e.g., it inhibits Suv39h1 methyltransferase degradation and enhances its activity. It can also modulate the activity of the p300 histone acetyltransferase [106,107,108]. In turn, SIRT6 deacetylates mainly H3K9 in the promotor regions of genes involved in metabolism [109]. It is also suggested that by deacetylating H3K9 in telomeric regions it can protect the cells from telomere dysfunction [110].

### 3.4. Non-Coding RNA

MicroRNAs are small (21–25 nucleotides) RNAs, which bind to complementary nascent mRNAs and trigger their degradation leading to inhibition of expression of specific genes. It has been estimated that 1%–4% genes in the animal genome encode miRNAs [111] and computational analysis predicts that individual miRNA can target approximately 200 transcripts [112]. It is known that miRNAs play a role in lifespan regulation [113].

### 3.5. Nuclear Architecture

Higher order 3D chromatin architecture involves containment of certain chromatin regions inside particular domains, called topologically associated domains (TADs). These provide favorable environment for gene expression regulation via enhancer-promoter interactions. TADs are enclosed by CTCF and cohesin that insulate them from the neighboring domains [114]. Chromatin can be also assigned either to so called compartment A that consists of transcriptionally active, gene-rich DNA regions or compartment B that overlaps with lamina associated domains (LADs) and is represented mostly by heterochromatin [115]. LADs are regions of heterochromatin enriched with H3K9me3 and H3K27me3 that tightly interact with nuclear lamina to organize chromosomes and stabilize the nuclear architecture [116].

**Figure 2:**
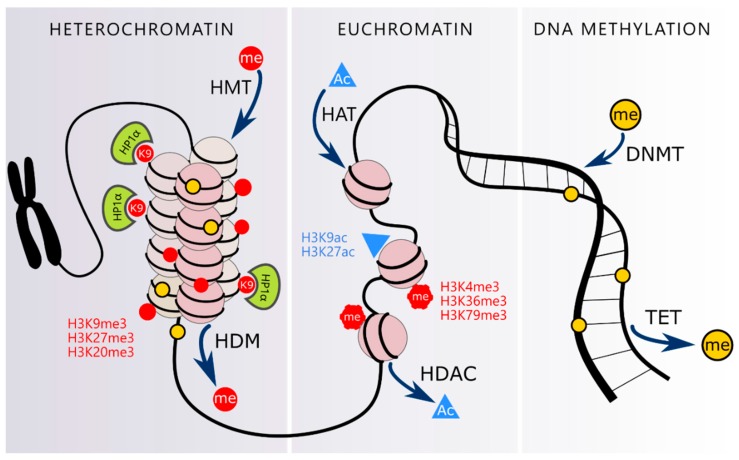
Epigenetic mechanisms shaping chromatin. Chromatin structure is precisely controlled by DNA methylation and post-translational modifications (PTMs; methylation, acetylation, etc.) deposited onto histone tails. Incorporation of methyl and acetyl group is guided by histone methyltransferases (HMTs) and histone acetyltransferases (HATs), while removal of these residues is achieved by histone demethylases (HDMs) and histone deacetylases (HDACs and Sirtuins). The action of these enzymes determines chromatin arrangement and affects gene transcription. Chromatin can adopt two distinct conformations, i.e., heterochromatin and euchromatin. Heterochromatin is a condensed and transcriptionally inactive structure, maintained mainly by methylation of lysine residues. Typical modifications include H3K27me3, H3K20me3 and H3K9me3. Euchromatin represents an open and active conformation. This structure is abundant in acetylated histones H3 and H4, and H3K4me3, H3K36me3 or H3K79me3. On the DNA level, gene expression can be regulated by methylation of cytosine residues by DNA methyltransferases (DNMTs) and oxidation of methylated cytosines by TET enzymes.

Mechanisms responsible for establishing and maintaining the chromatin structure are summarized in Figure 2.

### 3.6. Nuclear Landscape in Cell Senescence and Ageing

In ageing, the DNA methylation pattern is altered. The most clear alteration is related to a global decrease in DNA methylation observed in different species such as mouse, rat, cow, hamster and humans [117,118,119]. It has been demonstrated that the expression of DNMT1 (responsible for global DNA methylation) and DMNT3a decrease during ageing entailing a decline of DNA methylation [120]. However, gene-specific methylation of promoters is elevated, as it has been observed e.g., estrogen receptor and insulin-like growth factor-2 (IGF-2) [72,101,121].

Senescent cells are characterized by global loss of canonical histones both nucleosome forming (a “loss” of nucleosomes) and linker histones [122], which may occur due to histone proteins’ downregulation caused by cell-cycle arrest. A comparative quantitative analysis of global histone protein expression revealed a reduction in histone H3 and H4 content of about 30% and a 40% reduction in the turnover of H3 and H4 in senescent cells [123]. The loss of histones may also be a result of the induced autophagy of cytoplasmic chromatin fragments (CCFs) [124]. Senescent human fibroblasts form CCFs that contain DNA, γH2AX and repressive histone marks (mainly H3K9me3 and H3K27me3) [125], which are then digested in the lysosomes [126]. This leads to increased genome instability in ageing and senescence. On the other hand, histone variants are observed to accumulate with senescence [127]. In replicatively senescent and DNA damage-induced senescent mouse fibroblasts there is a significant increase in histone variant H2A.J as compared to proliferating cells [128]. Accumulation of H2A.J may promote expression of inflammatory genes contributing to SASP. Moreover, due to DNA damage, also γH2AX accumulates in senescent cells, as a part of DNA damage response machinery (DDR) [129]. In turn, prolonged DDR may lead to degradation of G9a and GLP methyltransferases causing reduction in H3K9me2, which is a part of constitutive heterochromatin [130]. Reduction in H3K9me3 in promoters of IL-8 and IL-6 encoding genes can be then compensated by acetylation of H3K9 and H4K16 leading to SASP. As shown by Hayakawa et al., senescent cells exhibit a gradual increase in the acetylation of H3K9 and H4K16 with the simultaneous downregulation of the histone deacetylase SIRT1 [131].

Generally, senescent cells exhibit global loss of HDACs and sirtuins [7,132] although certain genomic regions become enriched in acetylated histones. For example, senescent human fibroblasts show relatively elevated levels of H4K16ac in gene promoters. This impedes higher order chromatin packaging and results in an open chromatin structure in senescent cells that has been suggested to promote nucleosome exchange [133,134]. Moreover, studies have shown that H4K16ac is a prerequisite for the induction of H3K79 dimethylation while it acts as an inhibitor for H4K20me2 [135]. Contrarily, acetylation of K56 on H3 (H3K56ac) is reduced in replicatively senescent cells [135], probably due to SIRT6 activity [136]. It was shown, that SIRT6 also deacetylates H3K9ac at telomeric chromatin and maintains chromosomal stability [136]. The level/activity of majority of sirtuins decreases with age. It has been observed in many tissues and in many cell types in vitro (liver, arteries and vascular smooth muscle cells) [57,137,138]. One of the probable reasons of diminished sirtuins’ activity could be a reduction in NAD+ level.

On the other hand, during ageing, a global loss or redistribution of heterochromatin is observed, leading to increased genome instability and altered gene expression [15]. The loss of compaction is mostly due to both reduced methylation and usually increased acetylation of histones. Senescent cells are characterized by a drastic decrease in H3K9me3, and SUV39H1 methyltransferase, HP1α and H3K27me3 along with EZH2 methyltransferase. A similar trend of H3K9me3 and H3K27me3 loss is also observed in normal human ageing and premature ageing diseases, such as Hutchinson-Gilford progeria or Werner syndrome, suggesting that global loss of heterochromatin may be a common feature of ageing [139,140]. Oncogene-induced senescent (OIS) cells, in contrast, tend to have increased levels of repressive histone marks (H3K9me3 and H3K27me3) in comparison to non-senescent proliferating controls [141]. Moreover, the perinuclear heterochromatin associated with LAD reorganizes from the pre-existing heterochromatin into internal senescence-associated heterochromatin foci (SAHF) [142]. SAHFs are enriched in chromatin-associated proteins, namely HP1, HMGA and macro Histone 2A (mH2A). Additionally, SAHFs have a distinct layered structure: the core area is enriched in H3K9me3 [143], the periphery is abundant in H3K27me3 [144], while active chromatin marks can usually be found on the outside (e.g., H3K36me3) [145]. What is interesting, Chandra et al. revealed, that despite depletion of H3K9me3 and H3K27me3, OIS cells are still able to form SAHFs [144]. The downregulation of Lamin B1 and Lamin B Receptor (LBR) in OIS cells disrupts nuclear envelope structure and, as a result, disables heterochromatin anchorage. Moreover, recent studies show that the increased nuclear pore density in OIS contributes to SAHF formation [146]. The space beneath the nucleoporin complex is naturally depleted of heterochromatin; hence it shifts from the perinuclear to the internal space [147].

It is believed that an impact on chromatin compaction (forced induction of local chromatin condensation) could be sufficient to induce cell senescence due to its influence on the activation of the DDR pathway (ataxia telangiectasia mutated (ATM)- and ATR-dependent), which is not associated with DNA damage [148]. On the other hand, chromatin condensation is an integral part of the normal course of the DDR pathway and failure of this step results in impaired activation of this pathway. Moreover, downregulation of p300 histone acetyltransferase activity was sufficient to induce cell senescence [149].

MicroRNAs and their shuttles (extracellular vesicles in particular) regulate a large number of cellular functions and physiological processes and are currently recognized as regulators of healthy and unhealthy ageing [150]. In particular, the role of miRNAs is documented for brain functions in ageing [151,152,153]. For example, miRNAs control the level of two proteins, amyloid precursor protein (APP) and membrane-bound β-site APP-cleaving enzyme 1 (BACE1), which contribute to the formation of amyloid plaques in AD [154]. In a variety of ageing-related pathologies the miRNAs, similarly to other epigenetic regulators, are dysregulated [155,156]. The function of miRNAs in the regulation of the lifespan can be both inhibitory and activatory. In experimental studies, miRNAs were able to extend or shorten the lifespan of model organisms, such as C. elegans or mice [113,157,158,159]. For example, the loss of function mutation in the *C. elegans* miRNA *lin-4* dramatically reduced the lifespan of the nematode and overexpression of this miRNA extended it [160]. The loss of function mutation in miRNA *lin-14*, elongated lifespan, which is disturbed by the overexpression of this type of miRNA. Both these miRNAs, *lin-4* and *lin-14,* regulate the insulin/IGF-1 pathway. Other miRNAs, such as *miR-375*, which regulates insulin secretion in murine pancreatic islet cell [161] or *miR-122* in murine liver, are related to this signaling pathway [162]. Some miRNAs that target genes encoding proteins and enzymes belonging to the nutrient sensing pathways can act as novel therapeutics. They are involved in diabetes as well as ageing, by regulating inflammation [150] and, therefore, may play key role in the modulation of the ageing process.

Chromatin structure is also influenced by changes in the nuclear envelope. The inner surface of the nuclear envelope is lined with two types of lamins—A and B. Senescence is associated with the depletion of lamin B1 and mutations in the lamin A gene resulting in accumulation of defective prelamin A [163]. This leads to an abnormal nuclear morphology and chromosomal aberrations. Prelamin A was shown to reduce the import of DNA repair factor 53BP1 by mislocalizing nucleoprotein NUP153 [164], which results in persistent DNA damage. In HGPS, progerin (untruncated prelamin A) anchors to the inner nuclear membrane and reduces the attachment of H3K9me3 and H3K27me3 [165]. Heterochromatin shifts from nuclear lamina (a filamentous protein meshwork created by lamins and inner nuclear membrane proteins) to the center of nucleus (like in OIS).

**Figure 3:**
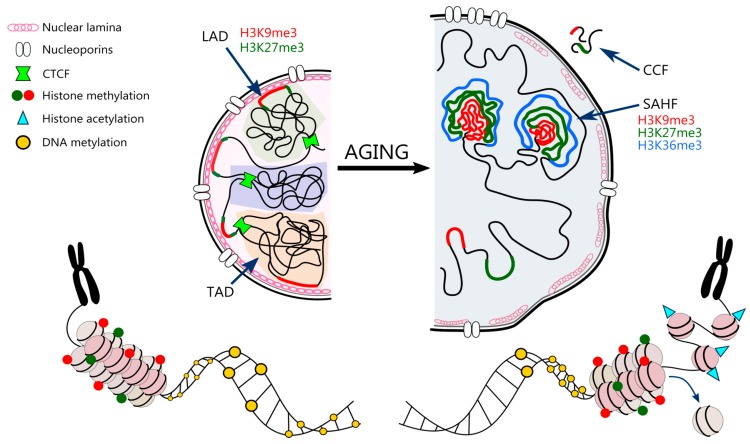
Nuclear and chromatin architecture changes in ageing and senescent cells. The nucleus of a proliferating cell contains a highly condensed chromatin enriched in methylated histones and hypermethylated DNA. The chromatin is compartmentalized into topologically associated domains (TADs) enclosed by CTCF protein and cohesin (not shown). Heterochromatic and gene-poor regions, characterized by the presence of H3K9me3 and H3K27me3, are in close contact with nuclear lamina (NL) forming lamina associated domains (LADs) contributing to the overall stabilization of the nuclear structure. During ageing/senescence DNA becomes globally hypomethylated with exception of promoters of particular genes, which may be hypermethylated. As a result, the gene expression pattern is significantly altered. Similarly, nucleosomes display a general loss of histones leading to loosely compacted euchromatin. Reduced compaction is also a result of accumulation of acetylated residues on histone tails and reduction of their methylation. OIS cells exhibit local condensation and redistribution of heterochromatin in the form of SAHFs. SAHFs emerge from detachment of heterochromatin from the nuclear lamina (due to downregulation of Lamin B1 and accumulation of dysfunctional prelamin A and progerin) and have a layered structure with a H3K9me3 rich core encircled with H3K27me3. On the outside, fragments of active chromatin are found (H3K36me3). Moreover, the local accumulation of nucleoporins prevents deposition of heterochromatin near the nuclear envelope. Disturbances in NL structure also result in cytoplasmic chromatin fragments that contain DNA and repressive histone marks.

All age/senescence-related changes in chromatin structure and nucleus architecture are summarized in Figure 3.

### 3.7. Epigenetics in Age-Related Diseases

An increasing amount of evidence derived from both clinical and experimental studies indicates that epigenetic deregulation, especially of DNA methylation, is frequently associated with ageing and may underlie the etiology of chronic diseases e.g., diabetic complication, CVD (atherosclerosis), cancer, metabolic disorder and neurodegeneration [166,167,168,169,170]. In atherosclerotic plaques isolated from human aorta a specific DNA methylation profile was observed. Compared to the healthy controls, several hypermethylated genes associated with endothelial and smooth muscle functions were found [166]. Moreover, DNA methylation may represent a useful biomarker for a disease risk, e.g., increased global DNA methylation, which was observed in peripheral blood leukocytes (PBL) of Singapore Chinese, is positively correlated with increased incidence of CVD, hypertension, diabetes and obesity [169]. There are much more examples linking epigenetic modifications with adiposity and diabetes. It has been shown that the methylation status of the promoters of genes encoding the retinoid X receptor alpha (RXRA) and endothelial nitric oxide synthase (eNOS) in umbilical cord tissue in healthy newborns, and peroxisome proliferator-activated receptor g coactivator-1a (Pgc-1a) in the blood of children is associated with adiposity in childhood [171,172]. Epigenetic analyses performed at different phases of the human life could be a predictor of the individual vulnerability to later obesity and metabolic disease. The ability to reverse the epigenetic mark would be invaluable for the treatment of certain diseases. Epigenetic alteration was demonstrated to be related to age-associated organ dysfunctions as in the case of renal ageing [173] and in ageing-related decline of immune response reviewed in [174]. The impact on immune response is achieved, on the one hand, via the alteration of epigenetic decoration of promoters and enhancers of regulators of fundamental immune cell functions. On the other hand, it is related to the epigenetic regulation of the inflammatory state. For example the induction of inflammatory cytokines (IL-8, MIP-1α- macrophage inflammatory protein 1-alpha) was caused by the loss of H3K9 methylation at the promoter regions [175]. Moreover, it has been shown that an increased age-related genomic instability was associated with the loss of heterochromatin marks (H3K9me3) and decrease in Suv39H1 expression [176].

However, the role of epigenetics in ageing is much more complex and is not limited only to one generation [177]. It has been demonstrated in a mouse model that advanced age increases the susceptibility for disease in offspring. The offspring of aged fathers had an exacerbation of age-associated phenotypes, reduced lifespan and were more prone to age-related pathologies than animals sired by young fathers. This was accompanied by numerous epigenetic alterations in the paternal germ line and offspring tissue, which manifested themselves by altered activation states of longevity-related cell signaling. Genome-wide epigenetic studies have revealed differences in gene promoter methylation, which was enriched in the case of genes involved in regulation of longevity pathways, in DNA from sperm of aged males and tissues from old father offspring (increased activity of mTORC1). This is an excellent example to demonstrate the role of epigenetics in the inheritance of ageing-related changes acquired during the lifetime. Furthermore, such results provide a very convincing reason to develop strategies in order to avoid adverse effects in the case of an advanced age of the parents.

## 4. Impact of the Diet on Chromatin Structure and Gene Expression

The consequences of a diet for health can be observed on a long-term basis, counted even in decades. Diet has an impact on the onset of ADR and longevity, but some effects can be effectively counteracted/eliminated by certain approaches [63]. Epigenetic mechanisms mediated by the nutritional effect, but also other environmental factors, establish a certain type of cellular memory. Such modifications can be copied by cells during cell division and define gene expression in new generation of cells, without changing their primary DNA sequence. As it was described in the previous paragraphs, the involvement of chromatin structure changes in senescence and ageing is significant; therefore, the protective approaches or chromatin rejuvenation approaches are considered as potential tools of anti-ageing interventions. Structural changes are, on the one hand, associated with a higher vulnerability to DNA damage and, on the other hand, with altered gene expression. The products present in the diet are able to modulate chromatin structure and, in this manner, regulate protein composition. There is a large number of data showing the impact of diet on DNA methylation on the expression of particular genes in animal models reviewed in [19]. Some data are present also for humans. One of the most spectacular results concerns differences in DNA methylation in individuals exposed peri-conceptually to famine during the Dutch Hunger Winter [178,179]. This caused a decreased methylation of e.g., *IGF-2* gene. Moreover, peri-conceptual folic acid supplementation influenced methylation of *IGF-2* promoter [180]. Dietary restriction, one of the currently known anti-ageing interventions to be used in humans, also acts by changing DNA methylation [181]. DR is generally strongly protective against age-related changes in DNA methylation [181]. This is associated with decreased p16 methylation and increased activity of DMNT1 (potential reversal of the global hypomethylation) [182,183,184]. There is also growing evidence that the epigenome is much more malleable at an early stage of development (pre- and postnatal development), however modifications are possible in each period of life. In adulthood the plasticity of epigenome also exists, reviewed in [19]. It is believed that epigenetic modifications can be potentially reversible and it makes this feature an attractive target for interventions [185].

Nutrients directly regulate both the transcription and translational processes and interfere with metabolism by affecting DNA methylation, histone modifications and post transcriptional gene regulation by non-coding RNAs. High fat, low protein or energy restricted diet can alter the epigenetics marks [182,186,187].

### 4.1. Impact of Nutrients on DNA Methylation

Nutrition may affect both the establishment and maintenance of the DNA methylation pattern, which can exert long-lasting health effects. Gene expression can be modulated by both nutrient and non-nutrient dietary compounds. Currently, the major interest is focused on the effects of specific micronutrients and non-nutritional dietary components. To this group belong natural compounds, e.g., polyphenols, the most abundant phytochemicals in fruits, vegetables and plant-derived beverages [16]. There is a growing evidence that polyphenols can control epigenetic changes and it is believed that they display the ability to reverse epigenetic modifications related to some pathologies, such as metabolic disorders, cardiovascular and neurodegenerative diseases and certain types of cancer. Therefore, such compounds as flavonoids, curcuminoids and stilbenes are promising in both disease prevention and intervention because they can modify gene expression with consequences for disease risk and health maintenance.

Nutritional and dietary factors have been postulated to affect DNA methylation by changing the availability of the methyl donors and altering the activity of the DNMT enzymes. To the first group belong micronutrients which are co-factors for enzymes involved in one-carbon metabolism, including folate, vitamin B6, vitamin B12, choline and methionine and those which can affect one-carbon metabolism indirectly [63]. The dietary selenium caused an imbalance in the methylation cycle by decreasing homocysteine concentration and, in consequence, reducing its availability for the methionine cycle. It led to reduced global DNA methylation in rat [188]. Modulators of DNMT activity include: selenium, genistein, quercetin, curcumin and green tea polyphenols, e.g., EGCG (epigallocatechin-gallate), whose effect has been documented mostly in cancer cells [189,190,191,192] and apigentin and resveratrol [193]. Flavonoids from tea and fruits are able to reverse hypermethylation and, in this manner, to reactivate tumor suppressor genes [194]. Selenite inhibited the binding of AP-1 transcription factor to DNA, which, in turn, reduced DMNT1 association with DNA (decreased methylation) [195,196] and selenium inhibited DNMT expression [197]. EGCG can inhibit DNMT1 directly by fitting to the binding pocket [194,198]. Quercetin is able to inhibit cancer cell proliferation due to the demethylation of the promoter of the p16 gene, which results in an enhanced expression of this tumor suppressor [199]. A very good example of the link between nutrition, epigenetics and phenotype is the supplementation of maternal diet of agouti mouse (a retrotransposon inserted upstream of the agouti gene causes ectopic expression of agouti protein, resulting in yellow fur, obesity and diabetes) with folic acid (methyl donor) and cofactors (vitamin B_12_, choline and betaine) [200] or genistein [201]. DNA methylation of the retrotransposon in the vicinity of the agouti gene restores normal expression of the agouti gene, the visible marker of which is darker hair color in offspring. Moreover, supplementation with methyl donors or genistein of agouti females protects the offspring from obesity (one of the risk factor for diabetes and CVD), which is characteristic for these mice [201,202].

Gene regulation can be mediated by secondary DNA structures, e.g., G-quadruplexes (G4) [203]. It is believed that these highly stable tetra-stranded structures, resolved by DNA helicases, are a causal factor in disorders following mutations in DNA helicase genes and are related to the phenotype of premature ageing. Such G4 structures are subjects of dynamic DNA modifications, which can affect genome stability and integrity, and alter gene expression. It is proposed that dietary nutrients, such as folate and antioxidants, can stabilize G4 structures [204].

In some situations, the application of phytochemicals, which alter DNA methylation, can lead to adverse epigenetic changes. Such observation has been made in the case of premenopausal women receiving isoflavones (to which genistein belongs) in every day diet [205]. This caused increased methylation of the promoters of tumour suppressor genes. On the other hand, genistein increased expression of cell cycle inhibitors, which can reduce the proliferation potential of cancer cells [206,207].

### 4.2. Impact of Nutrients on Histones Modifications

There are a lot of data showing that histones can be modified by nutrients and dietary compounds. It is related to the inhibitory potential of some dietary constituents interacting with enzymes involved in histones decoration. Histones marks can be altered by changed abundance and/or efficacy of the enzymes involved in their decoration or by impaired availability of the enzyme substrate. As it was mentioned before the chromatin status is controlled by HATs, HDACs, HMTs and HDMs. However, histones are not only methylated and acetylated. Histones tails can be modified by phosphorylation, ribosylation, ubiquitination, sumoylation and biotinylation [63], which visibly shows the complexity of the regulatory mechanism. To HDACs class III belong sirtuins, which are named “the enzymes of youth”, reviewed in [7]. The level of sirtuin decreases with age and the restoration of their level leads to rejuvenation.

Numerous inhibitors of HDACs are recruited from polyphenolic compounds. A lot of data are related to sirtuins, especially 1 and 6 [193]. However, HDACs inhibitors appear to be nonselective. Such inhibitors have shown beneficial effects in neurodegeneration, cancer, and inflammatory disorders. HDACs can be inhibited by butyrate (a short-chain carboxylic acid produced in the colon by bacterial fermentation of carbohydrates) and some polyphenols present in garlic, soybeans (e.g., genistein), garcinol and cinnamon [63,193]. To HDAC1 inhibitors belong quercetin, green tea polyphenols e.g., EGCG, luteolin and genistein [190,208,209,210]. Curcumin, a natural polyphenol, has been reported to function as both HDAC and HAT inhibitor [211,212] and is recognized as a hypomethylating agent [192]. Moreover, curcumin, dependently on the concentration, can act as sirtuin inhibitor (cytostatic concentrations) or activator (concentration which do not impair proliferation potential) [44,57,213]. Similar function is ascribed to EGCG, which influences enzymatic activity and expression of HATs, HDACs (HDAC1, 2, 3), and sirtuins [193,214,215], and for quercetin [216,217]. In turn, genistein increased HAT and decreased HDAC activity, including SIRT 1, and in this manner alters DNA accessibility [193,206]. HATs can be inhibited by green tea polyphenols, including EGCG, quercetin and by copper [214,216,218], and HMTs by EGCG [219]. Reduced availability of methyl donors in the diet also contribute to reduced HMT activity [220]. Posttranslational histones modification can be also shaped by resveratrol and catechins, i.e., other dietary polyphenols [16].

The fact that sirtuins exert multiple beneficial effects in the organism, such as reduction of the symptoms of CVD, diabetes and neurodegeneration as well as are linked to longevity, [221], it is reasonable to look for factors, which can increase their expression or activity. The latter can be enhanced by certain natural and synthetic compounds. To the first group belong flavones, stilbenes, chalcones, and anthocyanidins, which directly activate particular sirtuins in vitro. Such activity was shown for quercetin, butein, curcumin, fisetin, kaempferol, catechins, resveratrol, cilostazol, paeonol, daidzein and piceatannol [7,193,222,223]. Activation of sirtuins was also observed following mild physical activity [224,225,226]. The role of sirtuins in ageing and disease has been discussed in details by us in Grabowska et al. [7].

HDAC inhibitors can be one of the most promising prevention strategies concerning epigenetic changes including reduction/reversal of the already existing detrimental alternations. The restoration of the transcription of genes involved in proper metabolism, which decreases with age, can delay the functional decline of the organism [227]. Indeed, some examples have shown that inhibitors of HDACs are potent in extending the lifespan. This has been shown for fruit fly e.g., after feeding with 4-phenylbutyrate (PBA) [228] or with other HDAC inhibitors [229,230]. A global increase in histone acetylation and altered gene expression (e.g., of SOD, superoxide dismutase, cytochrome P450, glutathione S-transferase) significantly increased the lifespan. HDAC inhibitors were proposed in treatment of type II diabetes, ageing-associated cognitive decline and neurodegenerative diseases, such as AD and PD [231,232,233,234,235].

Some limitations are imposed by the lack of specificity of HDAC inhibitors. This poses a real problem since inhibition of most HDACs is considered as beneficial, while sirtuins have to be activated to exert their anti-ageing/anti-ARD role. To this aim, careful studies to find out some unique compounds to be involved in the selective regulation of HDAC activity are necessary.

### 4.3. Impact of Nutrients on miRNA Regulation

Polyphenols, e.g., EGCG, curcumin, resveratrol and quercetin, are also regulators of miRNAs, as has been shown in cancer cells [236,237,238,239,240]. EGCG decreased the expression of oncogenic miRNAs and increased the expression of tumour-suppressor miRNAs, which indicates it precise regulatory function [236]. Quercetin increased expression of miRNAs recognized as negative regulators of inflammatory genes [237]. The anticancer potential of genistein is also related to miRNA deregulation [238].

## 5. Impact of the Exercise on Gene Expression

The remodeling of the epigenome can be carried out by environmental factors other than nutrients, for example, by exercise. A large body of data indicates that physical activity (PA) can modulate gene expression through epigenetic alterations. This could be a beneficial strategy in chronic diseases, such as metabolic syndrome, diabetes, cancer (breast, colorectal and gastric cancer), cardiovascular and neurodegenerative diseases, reviewed in [65]. Physical activity remodels the skeletal muscle and adipose tissue [241,242,243,244]. It has been shown that six months of exercise led to the hypermethylation of *HDAC4* and *NCOR2* genes, which had an impact on the adipose tissue metabolism [244]. HDAC4 activity is related to repression of *GLUT4* transcription in adipocytes, and correlates with insulin resistance [245]. HDAC4 loss of function (export from the nucleus during exercise and loss of transcriptional repressive function) is also related to skeletal muscle adaptations to exercise; the latter effect has been interpreted as a result of activation of cellular *GLUT4* expression [246]. In turn, NCOR2, a nuclear co-repressor, is involved in the recruitment of, among others, HDAC4 [247]. It has been shown that alteration in the methylation pattern due to physical activity reduced the level of inflammatory mediators and prevented the occurrence of diseases associated with low-grade chronic inflammation [248]. PA acts as modulator of DNA methylation, histone modifications (particularly H3 and H4) and of miRNAs [249]. In the case of miRNAs, exercises modulate certain miRNAs, especially these involved in cancer, metabolic diseases and CVD [250,251,252]. Interestingly, it has been shown that, at the epigenetic level, exercises not only protect patients from type 2 diabetes but also, through maternal transmission of epigenetic modifications of genes involved in important metabolic pathways, have a beneficial impact on the offspring [253]. MicroRNAs regulate myogenesis, atrophy and sarcopenia [254]. During exercise a remarkable reduction of some types of miRNA, such as miR-107, miR-208b, miR-221 and miR-499, in mouse soleus muscle tissue was observed. The skeletal muscle biopsy of old people (68–72 years old) has shown overexpression or lower expression of numerous miRNAs in comparison with young individuals reviewed in [254]. MicroRNAs regulate two most important signaling pathways involved in ageing and longevity, namely the IGF axis and the mTOR pathway. It is documented that exercise can modify gene expression and activate signaling pathways, and that some of these changes are related to the expression of various miRNAs [255]. In this manner PA can strengthen exercise adaptation [256].

## 6. Autophagy as a Target of Nutritionally-Altered Epigenetic Pattern

Autophagy is considered as an anti-ageing catabolic process and is linked with/ cell survival and longevity [257]. It is one of the basic processes involved in intracellular degradation of damaged macromolecules and organelles that leads to self-cleaning and recycling within the cell. The regulation of autophagy is multistep and occurs at several levels. One of the mechanisms that regulate autophagy gene expression is the post-translational modification of histones, which control, for example, the nutrient sensor TOR (target of rapamycin), a factor involved in longevity. Autophagy is very often elevated in long-lived model organisms. SIRT1 and miRNAs are involved in this process. SIRT1 has been demonstrated to stimulate autophagosome formation and autophagic degradation resulting in increased autophagic flux [258]. While the SIRT1-mediated deacetylation of cytoplasmic proteins is required for autophagy induction, the activity of SIRT1 in the nucleus limits autophagy [257]. SIRT1 regulates autophagy gene expression through histone deacetylation. By deacetylating H4K16, it represses expression of various genes related to the early and late steps of autophagy (*Ulk1/ATG1*, *Ulk3/ATG1*, *Atg9A/ATG9*, *Lc3/ATG8*, *GabarapL2/ATG8*, and vacuolar membrane protein *Vmp1*). Histone H3 hypoacetylation (K9, K14, K18), via spermidine inhibition of histone acetyltransferase activity, increased expression of genes involved in autophagy such as *ATG7*, *ATG11*, *ATG15*. Moreover, methyltransferase EHMT2/G9a, by targeting H3K9, repressed transcription of autophagy genes [259]. Conversely, the downregulation of histone acetylation by KAT8 (HAT) (deacetylation of H4K16) induced autophagy. In this manner, by autophagy induction, SIRT1 exerts a protective effect in certain neurodegenerative disease models (e.g., PD) [260]. Autophagy can be regulated by acetyl coenzyme A (acetyl-CoA), which is a donor for acetylation reactions [257]. Reduced acetyl-CoA levels extended the lifespan of Drosophila [261]. The cationic polyamines (e.g., spermine and spermidine), whose level decreases with age in several tissues, and which are involved in autophagy regulation, have also an impact on histone acetylation status by regulating the activity of HATs and HDACs [257,262]. Spermidine inhibits HAT activity and leads to hypoacetylation of H3 on K9, K14 and K18. It has been shown that repressive marks H3K9me3, which accumulate with age, and H3K4me3 are linked to alterations in autophagy gene expression, as reviewed in [257]. The second manner of the epigenetic modulation of autophagy are miRNAs. For example, miR-34 is linked with autophagy and longevity. A decreased level of this regulator was observed in mice subjected to DR and it was correlated with increased lifespan [263]. Upregulation of miR-34 during ageing inhibits expression of the autophagy genes (e.g., *Atg9a*) [264]. MIR34 also leads to the inhibition of the expression of the SIRT1 [265] and mTOR pathway components. Several other miRNAs such as MIR376A, MIR376B, MIR181, MIR30D, MIR101 are related to autophagy inhibition, reviewed in [257].

## 7. The Microbiota Changes in the Elderly

The human body makes an excellent environment for a highly dynamic and complex ecosystem of bacterial archaeal, fungal and viral species, which is referred to as human microbiota. These microorganisms constitute lifelong symbiotic companions that are present from birth until our final days. The exact starting point of microbial colonization, however, is currently a subject of heated debate [266]. It was suggested, that the whole process may already start in utero [267,268], although recent studies return to the hypothesis that placenta is rather sterile, hence the fetus cannot acquire any microflora at this stage [269,270]. Nevertheless, inhabitation can surely be estimated to start at labor [271] and the initial composition of microbiota largely depends on the mode of delivery [272]. During birth, the first microorganisms start to colonize our skin, lungs, oral cavity and gastrointestinal tract. This process is relatively stabilized by the age of three to five [273]. Further development partly relies on the host genome [274] and gender [275] but also on a plethora of external factors, which include the type of diet [276,277], physical activity [278,279], the use of medication [280,281], and the living environment in general [282,283]. However, it should be noted, that the microbial composition is highly individual, hence the aforementioned modulatory factors might affect each host differently. Another factor significantly influencing the bacterial composition is ageing and age-related diseases [284]. It is generally accepted that as we age, the overall number and diversity of bacterial species decreases, often resulting in an imbalance between commensal bacteria in favor of pathobionts. This leads to further colonization with pathogens, production of inhibitory substances or toxins, and development of chronic inflammation [285]. Progressing dysbiosis contributes to detrimental health and age-related conditions such as cardiovascular diseases, neurodegenerative diseases (Alzheimer’s and Parkinson’s diseases), type II diabetes, chronic obstructive pulmonary disease (COPD), osteoporosis and different types of cancers. Moreover, alterations in human microbiota are also thought to be connected with allergies [286], obesity [287] and even depression [288,289]. Microbiota can modulate age-related changes related to frailty, such as innate immunity, sarcopenia, and cognitive functions [22]. Very extensive studies have shown that the differences in the composition of the gut microbiota between older people and younger adults are related to the gradual changes with time and not to chronological age [22].

### 7.1. Gut Microbiota

The largest and most prominent microbial reservoir is residing in the lower gastrointestinal tract (GIT). Gut microbiota play significant role in human health and diseases [290,291]. The microbiome is required for the digestion of complex plant polysaccharides, synthesis of short chain fatty acids (SCFA), indispensable amino acids and vitamins. Moreover, it neutralizes drugs and carcinogens, protects the host against pathogenic infection and regulates oxidative stress, as reviewed in [292]. There is some kind of interdependency and mutual relationship between microbiota and the host [293,294]. Microbiota can modulate immune cell functions, metabolism and insulin sensitivity. Moreover, the microbiome can regulate host gene expression through multiple mediators, such as SCFA, antioxidants and mediators of inflammation. Nutrition plays a pivotal role in this process because all mentioned mediators synthesized by gut bacteria, are derived from the diet [295].

There are about 1000–1500 recognized bacterial species living in the gut and each person harbors about 150 different species, which together contain more genes than the human genome and constitute a sort of a second genome [294]. GIT is the most susceptible site of age-related changes in the bacterial composition, connected with gradual decrease in intestinal barrier function [296], impaired nutrient absorption [297], increased risk of developing cancers [298,299] and brain disorders [300,301]. The most visible alteration of gut microbiota is the reduction in their diversity, shifts in the dominant species, which is related to a decrease in beneficial microorganisms and increase in facultative anaerobic bacteria. This results in a lower availability of short chain fatty acids, because different compositions of microbiota have an impact on the production of bacterial metabolites [17,284]. Age-related changes in the gut microbiota are also associated with alteration in the physiology of gastrointestinal tract, such as a decrease in saliva production and gastric acid secretion, lower absorption of nutrients (iron and vitamin B12), slower gastrointestinal motor function and, finally, altered diet composition associated with decreased food intake and a decline in the functioning of the immune system. This makes elderly people more prone to infections and frailty [17,302,303,304,305].

GIT is composed of small and large intestines. Small intestine is responsible for nutrient absorption, facilitated by metabolic action of microbial cells that are rather low in abundancy but stable in composition [306]. During ageing however, the incidents of the microbial overgrowth, namely Small Intestinal Bacterial Overgrowth (SIBO) may occur. This leads to the malabsorption of micronutrients and, as a result, to malnutrition, abdominal distension, diarrhea and flatulence [307]. Large intestine, on the other hand, is the most densely colonized part of the human body. The average number of bacterial colonies ranges from 10^11^ to 10^12^, making their genome nearly 150 times larger than a human [294]. Bowels are a place where the final fermentation and absorption of key nutrients take place. The most abundant phyla in homeostatic conditions are *Actinobacteria*, *Bacteroidetes*, *Firmicutes*, *Proteobacteria*, and *Verrucomicrobia*. Along with mucosal secretions, the bacteria form a rather thick and resistant biofilm, that line the epithelium, working as a mediator between the lumen and host’s body [308]. These commensal bacteria produce short-chain fatty acids (SCFA), synthesize vitamins K and B and protect the host form the external pathogens (production of bacteriocins). All of these compounds exert an immunomodulatory effect by stimulating immunoglobulin A production, down-regulating proinflammatory cytokines and inducting regulatory T cells [309]. In ageing however, everything is disturbed as there is a decline in the number and diversity of commensals [310]. *Bifidobacteria* and *Enterobacter* decrease in elderly, while *Bacteroidetes* increase in comparison to young adults [311]. Moreover, beneficial bacteria are usually overtaken by pathobionts that disrupt the epithelial integrity [312] and lead to secretion of inflammatory markers, such as IL-6 and IL-8, resulting in elevated (and persistent) inflammation [22]. Intestinal inflammation is characterized by thinner or even degraded inner mucus layer, enabling bacteria to reach the epithelium. Pathogenic strains produce exotoxins [313] that induce changes to tight junctions, leading to increased gut permeability, also defined as “leaky gut”. This, in consequence, contributes to irritable bowel disease [314], the development of colon cancer and may also lead to a disrupted blood-brain axis detrimentally affecting neurodegenerative diseases [315,316,317]. Gut permeability, however, can be modified by beneficial bacterial strains, such as *Lactobacillus plantarum*, *Escherichia coli*, Nissle, and *Bifidobacterium infantis*, which increase expression of proteins forming tight junctions, and as a result, strengthen the intestinal barrier [318]. Although bacteria have the leading role in the gut composition maintenance in the elderly, the methanogenic archaea also play important role in human ageing. The diversity of methanogens is increased in the elderly as compared to young adults [319], however there is no significant increase in prevalence. However, methane formed by these archea is more abundant in the elderly, which affects smooth muscle cells and results in slowed bowel transit [320]. This also impacts the intestinal pH. In the elderly, the pH is increased compared to young controls, and has deleterious impact on the beneficial bacteria.

### 7.2. Other than Gut Microbiota

Ageing has a tremendous influence on the microbial composition within all human body. For instance, human skin harbors fairly different colonies between dry volar forearm, quite moist axillary vault and sebaceous face [321], where the bacterial abundancy is extremely age-dependent [322]. In the elderly, higher species richness is noted in palms, forearms and forehead than in young controls, while young display greater diversity on nares, glabella or back [323]. With age, the total number of aerobes (especially *Propionibacterium*) on cheeks and forehead of healthy elderly decreases as compared to young adults [323,324], which tightly correlates with lower secretion of sebum [324]. However, in some skin conditions the sebum can be overproduced and lead to seborrheic dermatitis (SD), which is also a non-motor symptom of Parkinson’s Disease [325]. The skin of PD patients with SD have increased densities of fungi *Malassezia* spp. and bacteria *Streptomyces* spp., that produce complex volatile organic compounds, which in turn may serve as a noninvasive diagnostic tool of early Parkinson’s disease [326]. Similarly, breath analysis of microbial volatile organic compounds may also be used for diagnosis of Chronic Obstructive Pulmonary Disease (COPD) in the elderly [327]. Patients with COPD display significant increase in pathogenic Gram-negative bacteria [328] with commensurate decrease in diversity of resident bacterial communities [329,330] as compared to healthy subjects and young adults [331]. Elderly also exhibit profound changes in intraoral microflora [332], notably on the dental and subgingival plaque. These often result in inflammatory conditions like periodontitis, in which the rise in Gram-negative bacteria is observed [333,334]. The main pathogen of chronic periodontitis, *Porphyromonas gingivalis*, is a serious risk factor for developing Alzheimer’s disease [335].

## 8. Impact of the Diet on Microbiota

As mentioned earlier, the role of the microbiome is strongly associated with the pace of ageing and the quality of life of the elderly. Some data show that gut microbiota is implicated in the pathogenesis of metabolic disorders, such as diabetes and obesity, and promotes atherosclerosis, and vascular endothelial dysfunction [336,337,338,339]. On the other hand, the microbiome is also involved in atherosclerosis retardation by generating metabolites of dietary flavonoids, which stimulate reverse cholesterol transport in macrophages [340]. However, the most recognized impact of the microbiome is related to the symptoms of frailty [22,295].

The interaction between nutrients (micro and macro) and microbiota is very tight. Such a relation concerns, e.g., polyphenols [341,342,343,344]. The bioavailability of this group of compounds is very low. Gut microbiota metabolize them into active and bioavailable metabolites. On the other hand, polyphenols modulate microbiota because they can exert prebiotic-like effects and promote growth of some bacterial species (particularly hydrolysable and condensed tannins derived from polyphenol-rich foods, including cocoa, tea, grapes, wine, berries, pomegranates, and nuts). The impact is thus reciprocal [345,346]. Curcumin is also able to modulate the diversity of microbiota [341,342,343,347]. The composition of gut microbiota is very often the cause of some confusing results obtained from clinical trials, because of individual variability. It is suggested that the composition of bacteria is a key element in the response to polyphenol treatment and that gut microbiota are probably responsible for polyphenols’ health effects by regulating their bioavailability [345,348]. The bioavailability of most micronutrients is poor due to low absorption and fast metabolism leading to rapid elimination from the organism. One of the modifications related to the detoxication mechanism is glucuronidation, which has been well documented e.g., for curcumin, quercetin, genistein and ginger phenolics [349,350,351,352,353]. Mono- and diglucuronides are less active then free compounds [354]. One of the products of the gut bacteria is β-glucuronidase [355]. This enzyme is involved in hydrolysis of the glucuronide moiety [351,356], and has an impact on the level of free compounds.

Recent data have shown that SIRT1 can mediate the interaction between the host and the microbiome [357]. Mice with a deletion of SIRT1 in the intestinal epithelium exhibited increased activation of NFκB and inflammation and, moreover, had altered fecal microbiota due to altered bile acid metabolism. Additionally, the lower expression of SIRT1 was observed in intestinal tissues from patients suffering from colitis. It is suggested that SIRT1 prevents intestinal inflammation by the regulatory impact on gut microbiota. Similar role is attributed to SIRT3, the level of which decreases in colorectal cancer [358]. It interacts with gut microbiota, which plays a central role in the resistance to colon cancer, and in this manner acts as an anti-inflammatory and tumor-suppressing protein. On the other hand, SIRT1 can also be regulated by the microbiome [359]. The expression of SIRT1 is governed by microRNAs (e.g., miR-204) while expression of the latter is remotely controlled by the microbiome. The function of the endothelium is thus impaired since miR-204 downregulates the level of SIRT1 (antibiotics decrease miR-204 and increases Sirt1 expression). Additionally, sirtuins and AMPK can be regulated by SCFA produced by microbiota, reviewed in [292]. This can have an impact on chromatin structure and gene expression. SIRT1 is one of the proteins directly regulated by SCFA [360]. N-butyrate, activated several regulatory pathways including that involving AMPK signaling [361], and inhibited histone deacetylase (HDAC4), as was evidenced by the increased acetylation of H3K9 in the liver and brain [362]. A healthy gut microbiota produces significant amounts of folate and vitamin B_12_ [363], which are donors of the methyl group essential for DNA methylation. Moreover, gut bacteria synthesize amino acids, such as tryptophan, which stimulates the IGF-1/p70s6k/mTor pathway and in this manner activates gene expression [364]. An association has been documented between the intestinal microbiota composition and weight loss caused by CR. Moreover, it has been shown in animal models that CR-induced lifespan elongation was accompanied by a structural modulation of gut microbiota [365].

Maintaining a proper diet is important for individuals of all age. Moreover, the nutritional needs of elderly do not substantially differ from those of younger adults. However, it is particularly important to promote some nutritional strategies among those of older age because of the risk of malnutrition due to impaired absorption, loss of appetite and chewing difficulties, which may affect the proper nutritional status [366]. These strategies are elaborated to restore the diversity of microbiota in old people in order to reduce some adverse consequences of ageing, including frailty.

## 9. Conclusions

Well-recognized anti-ageing strategies have been for a long time linked to a proper diet. Recently, it has been well documented that diet can modify chromatin structure. Moreover, it was proven that certain epigenetic modifications are long-lasting and can be inherited while others can be quite easily reversed. Therefore, nutrients can determine our health and disease even if they were consumed a long time ago, e.g., in the prenatal period. They can play a pivotal role in the ageing process and longevity. To the puzzle composed of two elements, i.e., nutrients and epigenetics, the microbiome should also be added. The interaction of these three seemingly independent elements creates a coherent picture that may be useful for developing potential anti-ageing strategies. The awareness of this relationship allows the adoption of a holistic approach to ageing with only one aim in mind: to synchronize all strategies in order to optimize the conditions necessary to healthspan elongation. The age-related epigenetic changes can be reversed by some macromolecules from the diet, the bioavailability of which depends on the microbiome, which per se affects the age-related dysfunctions. The urgent issue to address is how the current knowledge can be transferred into practice. More broadly, it is a question of how to target the individual needs of people, based on their epigenetic history and the composition of the microbiome, taking simultaneously into account age and gender, and not neglecting the impact of environment, which modulates the epigenetic landscape. The current view suggests that the epigenetic age, defined as the presence of characteristic markers, could be a more powerful predictor of future health decline and ARD than chronological age. The most important scope for future studies is to develop the strategies and promote such dietary habits, which will be effective in modulating the epigenome (directly or by the impact on microbiome) to eliminate or delay the expression of genes associated with the initiation and progression of ARD and ageing disabilities (e.g., frailty). Such nutritional habits/strategies must be adapted to age. It is essential to identify and characterize the most potent dietary factors with a strong and defined impact on the epigenome and microbiome, and to recognize the mechanism of their action.
